# Supplementary material for: Multi‐dataset identification of innovative feature genes and molecular mechanisms in keratoconus
Source: J Cell Mol Med. 2024 Sep 19;28(18):e70079. doi: 10.1111/jcmm.70079 (PMC11412914; doi:10.1111/jcmm.70079)
Supplement: Supplementary file 2 — Material S2. [file JCMM-28-e70079-s002.zip › 7.GSEA analysis/1.ARL11/KEGG_LONG_TERM_DEPRESSION.html]

Details for gene set KEGG\_LONG\_TERM\_DEPRESSION[GSEA]

|  || Dataset | ARL11.group.cls#h\_versus\_l |
| Phenotype | group.cls#h\_versus\_l |
| Upregulated in class | l |
| GeneSet | KEGG\_LONG\_TERM\_DEPRESSION |
| Enrichment Score (ES) | -0.3711819 |
| Normalized Enrichment Score (NES) | -1.2521331 |
| Nominal p-value | 0.16796875 |
| FDR q-value | 1.0 |
| FWER p-Value | 0.973 |
Table: GSEA Results Summary

  

Fig 1: Enrichment plot: KEGG\_LONG\_TERM\_DEPRESSION      
 Profile of the Running ES Score & Positions of GeneSet Members on the Rank Ordered List

  

| SYMBOL | TITLE | RANK IN GENE LIST | RANK METRIC SCORE | RUNNING ES | CORE ENRICHMENT || 1 | PLCB2 | na | 134 | 0.619 | 0.0731 | No |
| 2 | GRM5 | na | 2391 | 0.271 | 0.0417 | No |
| 3 | CACNA1A | na | 2621 | 0.259 | 0.0673 | No |
| 4 | PLA2G4E | na | 4751 | 0.174 | 0.0275 | No |
| 5 | IGF1R | na | 5874 | 0.140 | 0.0126 | No |
| 6 | PLA2G3 | na | 6310 | 0.129 | 0.0160 | No |
| 7 | PPP1R17 | na | 6499 | 0.124 | 0.0260 | No |
| 8 | GNAI3 | na | 7891 | 0.095 | -0.0023 | No |
| 9 | PLA2G12B | na | 9215 | 0.072 | -0.0316 | No |
| 10 | ITPR2 | na | 9655 | 0.065 | -0.0362 | No |
| 11 | PRKG2 | na | 10052 | 0.060 | -0.0402 | No |
| 12 | ITPR1 | na | 10204 | 0.058 | -0.0374 | No |
| 13 | PLCB3 | na | 10228 | 0.057 | -0.0309 | No |
| 14 | PRKCA | na | 10617 | 0.052 | -0.0356 | No |
| 15 | BRAF | na | 10628 | 0.052 | -0.0294 | No |
| 16 | KRAS | na | 10630 | 0.052 | -0.0230 | No |
| 17 | GNAI2 | na | 13045 | 0.027 | -0.0893 | No |
| 18 | PLA2G2C | na | 13424 | 0.024 | -0.0973 | No |
| 19 | JMJD7-PLA2G4B | na | 14509 | 0.015 | -0.1267 | No |
| 20 | GNA11 | na | 15711 | 0.006 | -0.1606 | No |
| 21 | GNAS | na | 16257 | 0.002 | -0.1761 | No |
| 22 | MAP2K2 | na | 16997 | -0.003 | -0.1970 | No |
| 23 | PLA2G4A | na | 17245 | -0.005 | -0.2035 | No |
| 24 | PRKCG | na | 17545 | -0.007 | -0.2113 | No |
| 25 | PLA2G6 | na | 17692 | -0.008 | -0.2145 | No |
| 26 | PLA2G12A | na | 17822 | -0.009 | -0.2171 | No |
| 27 | GNAQ | na | 17828 | -0.009 | -0.2161 | No |
| 28 | ITPR3 | na | 18064 | -0.011 | -0.2215 | No |
| 29 | PLCB4 | na | 18133 | -0.011 | -0.2221 | No |
| 30 | IGF1 | na | 18329 | -0.013 | -0.2262 | No |
| 31 | HRAS | na | 18435 | -0.013 | -0.2275 | No |
| 32 | GRID2 | na | 19093 | -0.018 | -0.2443 | No |
| 33 | GNAZ | na | 19703 | -0.022 | -0.2592 | No |
| 34 | PPP2R1A | na | 19944 | -0.023 | -0.2632 | No |
| 35 | PLA2G2E | na | 19951 | -0.023 | -0.2604 | No |
| 36 | RYR1 | na | 20412 | -0.027 | -0.2704 | No |
| 37 | GRM1 | na | 21083 | -0.032 | -0.2857 | No |
| 38 | GRIA2 | na | 22338 | -0.043 | -0.3165 | No |
| 39 | MAPK3 | na | 22614 | -0.046 | -0.3187 | No |
| 40 | RAF1 | na | 23336 | -0.054 | -0.3328 | No |
| 41 | PLA2G10 | na | 23788 | -0.060 | -0.3384 | No |
| 42 | MAPK1 | na | 24626 | -0.071 | -0.3537 | No |
| 43 | PPP2CA | na | 25060 | -0.077 | -0.3566 | No |
| 44 | GNA12 | na | 25331 | -0.080 | -0.3544 | No |
| 45 | LYN | na | 25913 | -0.089 | -0.3601 | Yes |
| 46 | GRIA1 | na | 25975 | -0.090 | -0.3507 | Yes |
| 47 | ARAF | na | 26195 | -0.093 | -0.3454 | Yes |
| 48 | GNA13 | na | 26206 | -0.094 | -0.3341 | Yes |
| 49 | PPP2R1B | na | 26783 | -0.104 | -0.3378 | Yes |
| 50 | GNAI1 | na | 27299 | -0.115 | -0.3384 | Yes |
| 51 | PPP2CB | na | 27315 | -0.115 | -0.3245 | Yes |
| 52 | NOS1 | na | 27670 | -0.123 | -0.3194 | Yes |
| 53 | NRAS | na | 27790 | -0.126 | -0.3072 | Yes |
| 54 | PLA2G2D | na | 27985 | -0.130 | -0.2966 | Yes |
| 55 | GNAO1 | na | 28977 | -0.156 | -0.3058 | Yes |
| 56 | PLA2G2F | na | 30623 | -0.208 | -0.3274 | Yes |
| 57 | MAP2K1 | na | 31070 | -0.223 | -0.3125 | Yes |
| 58 | GUCY1A2 | na | 31473 | -0.238 | -0.2945 | Yes |
| 59 | PLA2G5 | na | 32093 | -0.264 | -0.2795 | Yes |
| 60 | PLCB1 | na | 32251 | -0.272 | -0.2502 | Yes |
| 61 | PLA2G2A | na | 32310 | -0.275 | -0.2177 | Yes |
| 62 | CRHR1 | na | 32913 | -0.316 | -0.1958 | Yes |
| 63 | CRH | na | 33019 | -0.323 | -0.1586 | Yes |
| 64 | PLA2G1B | na | 33526 | -0.362 | -0.1282 | Yes |
| 65 | PRKCB | na | 33863 | -0.399 | -0.0883 | Yes |
| 66 | PRKG1 | na | 33894 | -0.404 | -0.0389 | Yes |
| 67 | GRIA3 | na | 34427 | -0.506 | 0.0087 | Yes |
Table: GSEA details [plain text format]

  

Fig 2: KEGG\_LONG\_TERM\_DEPRESSION      
 Blue-Pink O' Gram in the Space of the Analyzed GeneSet

  

Fig 3: KEGG\_LONG\_TERM\_DEPRESSION: Random ES distribution      
 Gene set null distribution of ES for **KEGG\_LONG\_TERM\_DEPRESSION**
